# Supplementary material for: Intratumoral synthesis of nano-metalchelate for tumor catalytic therapy by ligand field-enhanced coordination
Source: Nat Commun. 2021 Jun 7;12:3393. doi: 10.1038/s41467-021-23710-y (PMC8184762; doi:10.1038/s41467-021-23710-y)
Supplement: Supplementary file 1 — Supplementary information. [file 41467_2021_23710_MOESM1_ESM.pdf]

Supplementary Information

Intratumoral Synthesis of Nano-Metalchelate for Tumor Catalytic Therapy by Ligand Field-Enhanced Coordination

Bowen Yang [1,2], *Heliang Yao* [1], *Han Tian* [1,2], *Zhiguo Yu* [1,2], *Yuedong Guo* [1,2], *Yuemei Wang* [1,2], *Jiacai Yang* [1,2], *Chang Chen* [1,2], *and Jianlin Shi* [1]*

[1] State Key Laboratory of High Performance Ceramics and Superfine Microstructure, Shanghai Institute of Ceramics, Chinese Academy of Sciences, Shanghai, 200050, P. R. China;

[2] Center of Materials Science and Optoelectronics Engineering, University of Chinese Academy of Sciences, Beijing, 100049, P. R. China.

E-mail: jlshi@mail.sic.ac.cn

Supplementary Figures

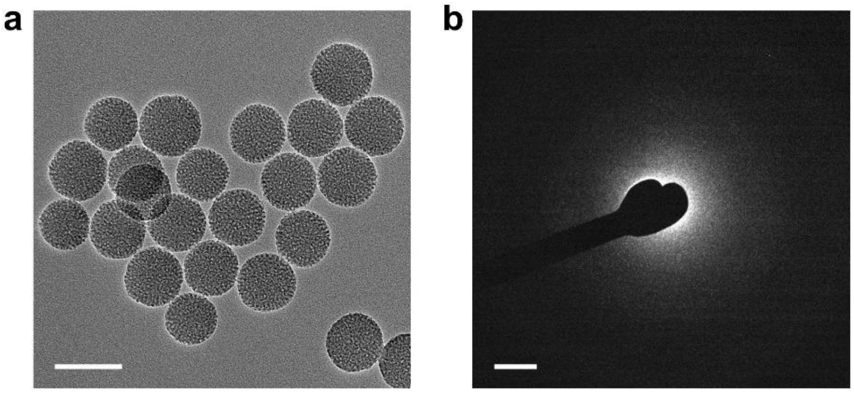

Supplementary Figure 1. TEM image **(a)** of MSNs and corresponding SAED pattern **(b)**. Scale bars, 100 nm and 2 nm^{-1}. A representative image of three replicates is shown.

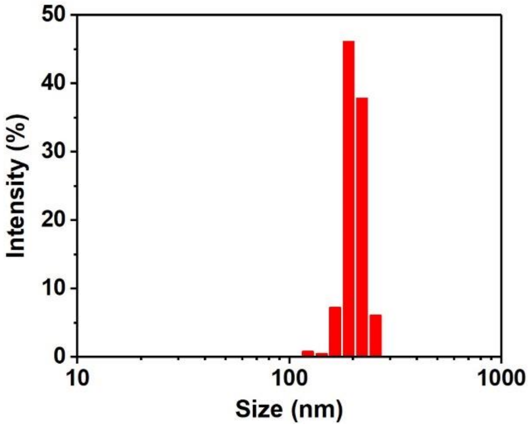

Supplementary Figure 2. DLS measurement of Fe-HMSNs indicating size distribution. Source data are provided as a Source Data file.

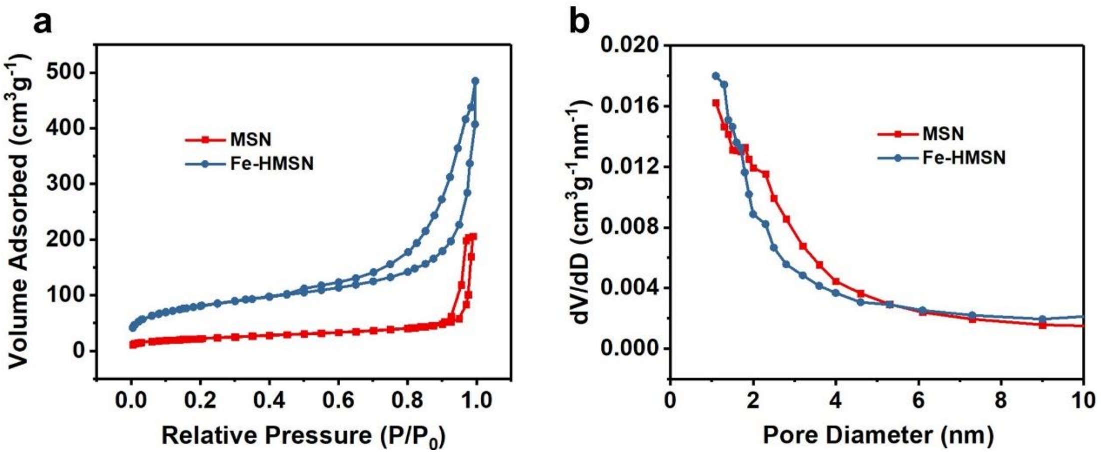

Supplementary Figure 3. N_2 adsorption-desorption isotherms **(a)** and pore-size distributions **(b)** of MSNs and Fe-HMSNs. Source data are provided as a Source Data file.

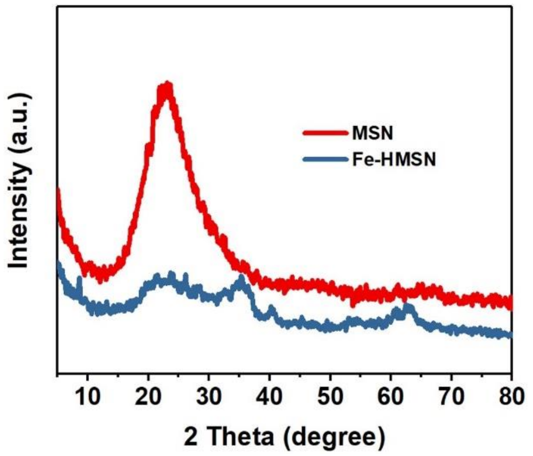

Supplementary Figure 4. XRD patterns of MSNs and Fe-HMSNs. Source data are provided as a Source Data file.

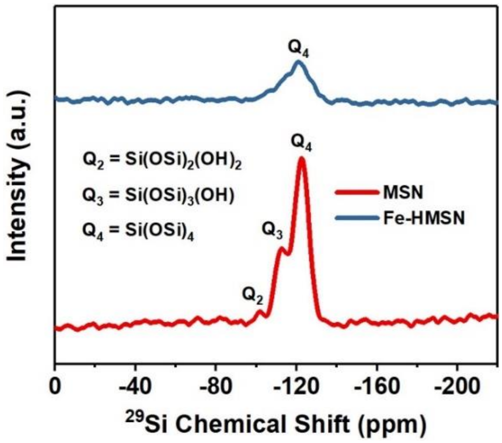

Supplementary Figure 5. ^{29}Si solid-state MAS NMR spectra of MSNs and Fe-HMSNs. Source data are provided as a Source Data file.

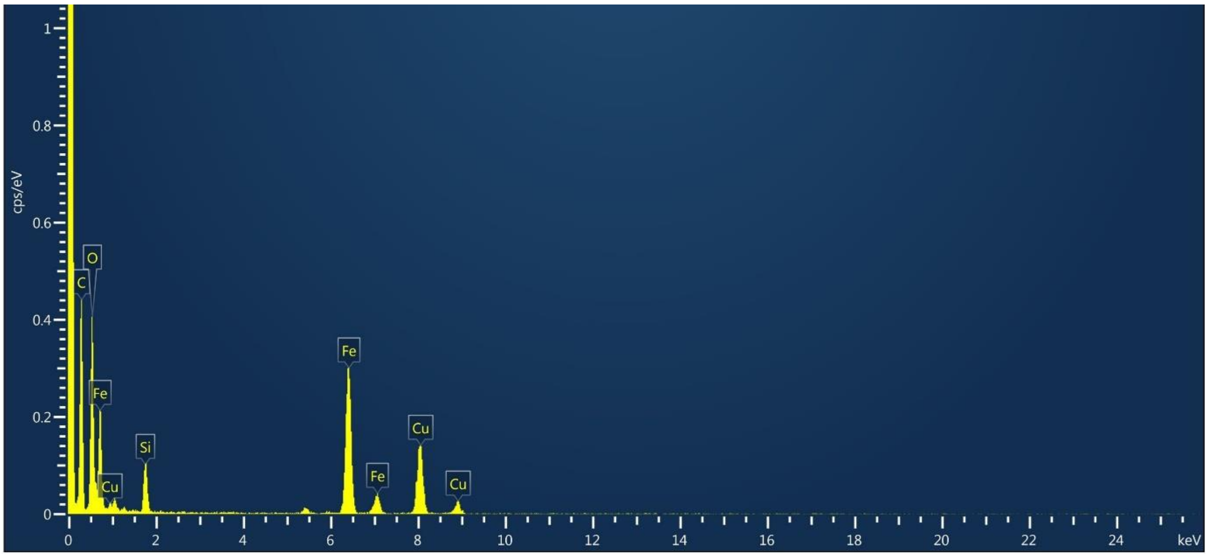

Supplementary Figure 6. EDS profile of Fe-HMSN sample on a cooper grid.

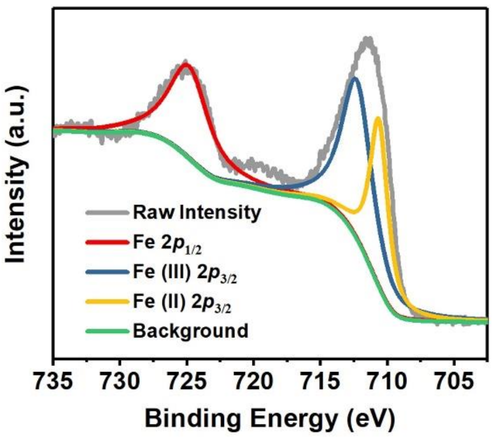

Supplementary Figure 7. Fe 2*p* spectrum of XPS spectra of Fe-HMSNs. Source data are provided as a Source Data file.

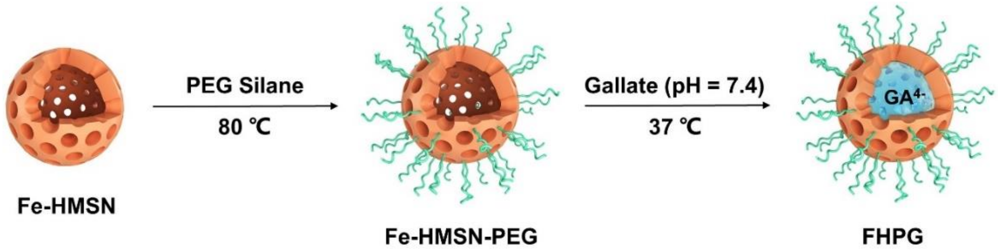

Supplementary Figure 8. Synthetic procedure of FHPG. Fe-HMSNs were first modified with PEG in a mild hydrothermal condition then loaded with gallate at a room temperature.

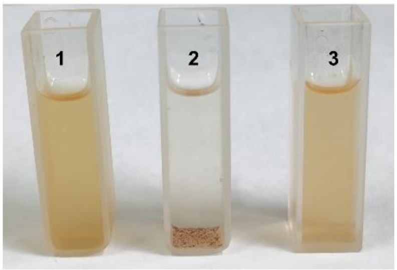

Supplementary Figure 9. Digital photo of Fe-HMSNs dispersed in deionized water (1) and PBS (2), as well as Fe-HMSNs-PEG dispersed in PBS (3).

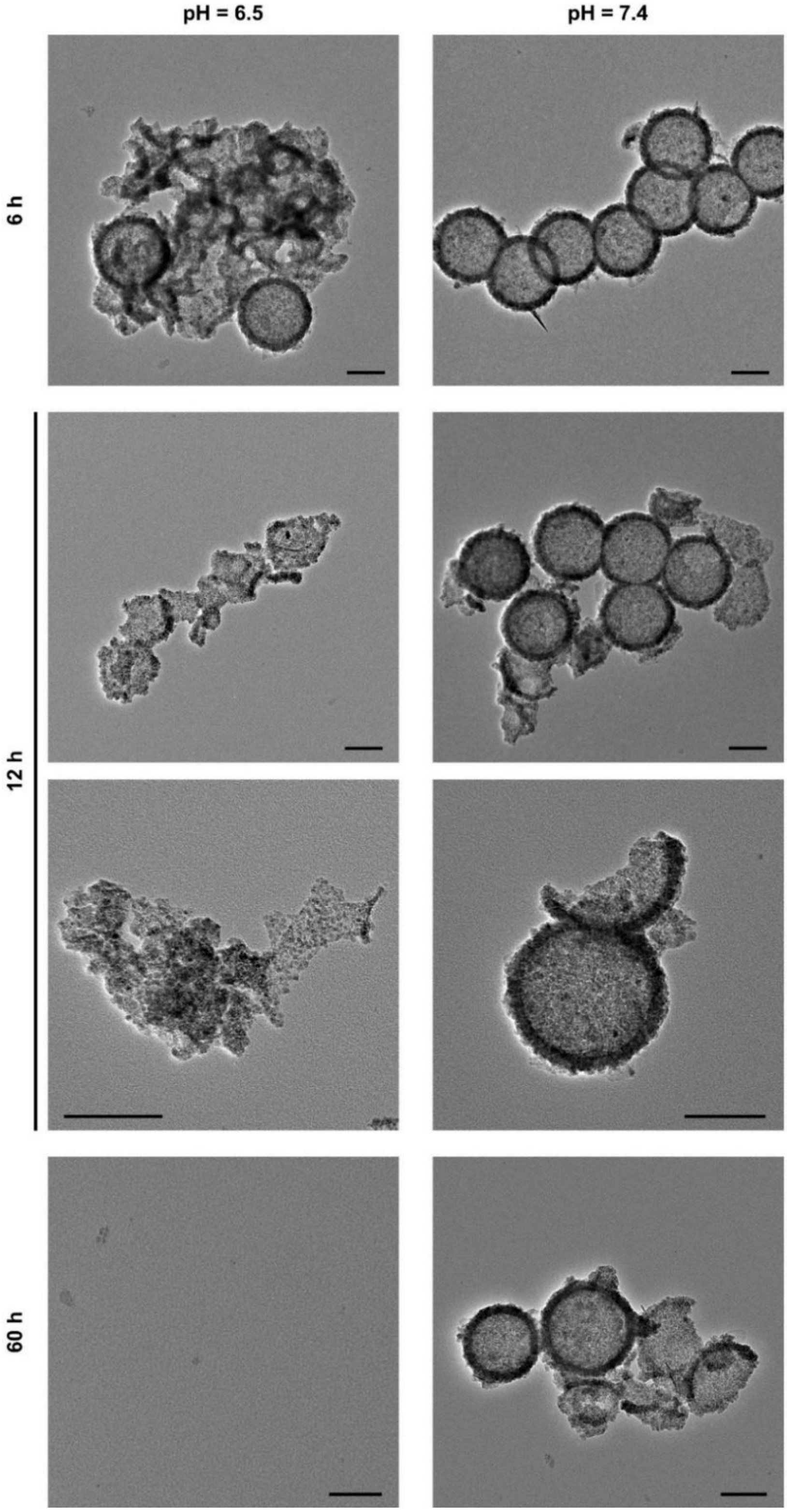

Supplementary Figure 10. TEM images of Fe-HMSNs-PEG after degradation in SBF of different pH values for 6, 12, and 60 h. Scale bars, 100 nm. A representative image of three replicates from each group is shown.

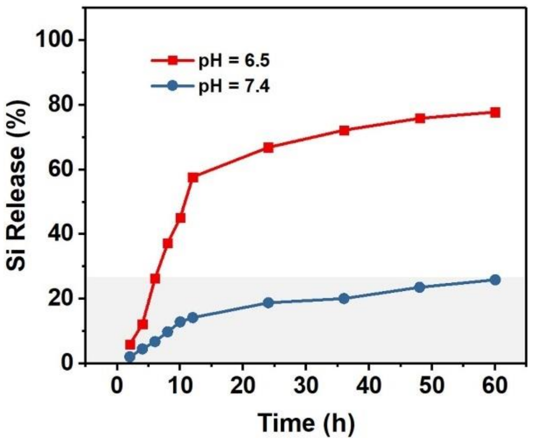

Supplementary Figure 11. Accumulated release profiles of Si element from Fe-HMSNs in SBF of different pHs. Source data are provided as a Source Data file.

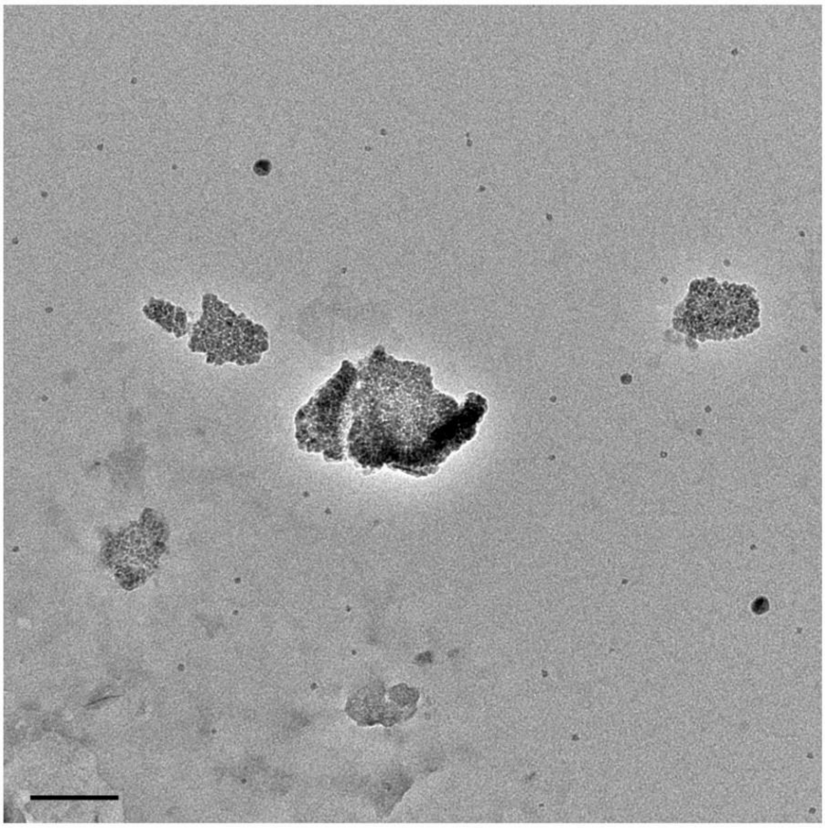

Supplementary Figure 12. TEM image of FHPG sample after degradation for 12 h in SBF (pH = 6.5). Scale bar, 100 nm. A representative image of three replicates is shown.

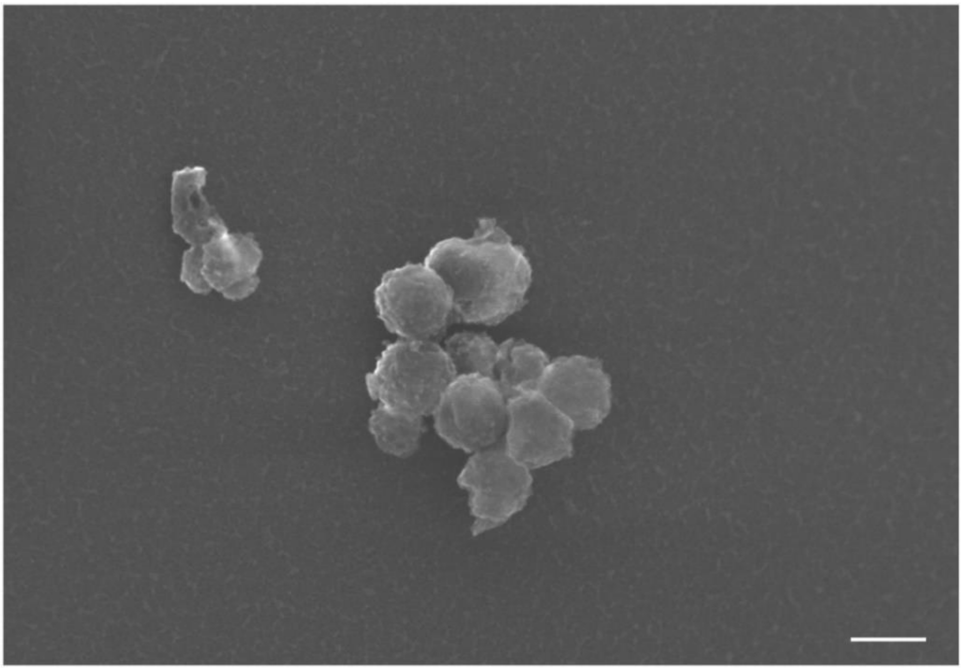

Supplementary Figure 13. SEM image of FHPG sample after degradation for 6 h in SBF (pH = 6.5). Scale bar, 100 nm. A representative image of three replicates is shown.

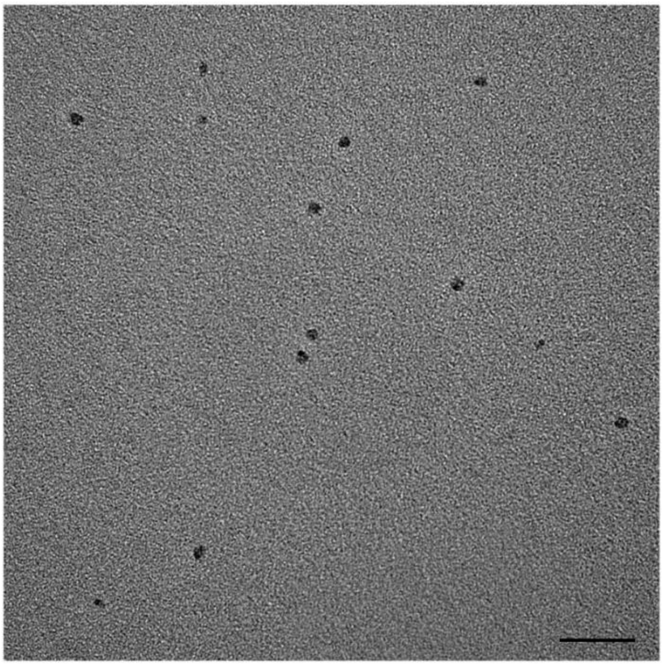

Supplementary Figure 14. TEM image of FHPG sample after degradation for 60 h in SBF (pH = 6.5). Scale bar, 50 nm. A representative image of three replicates is shown.

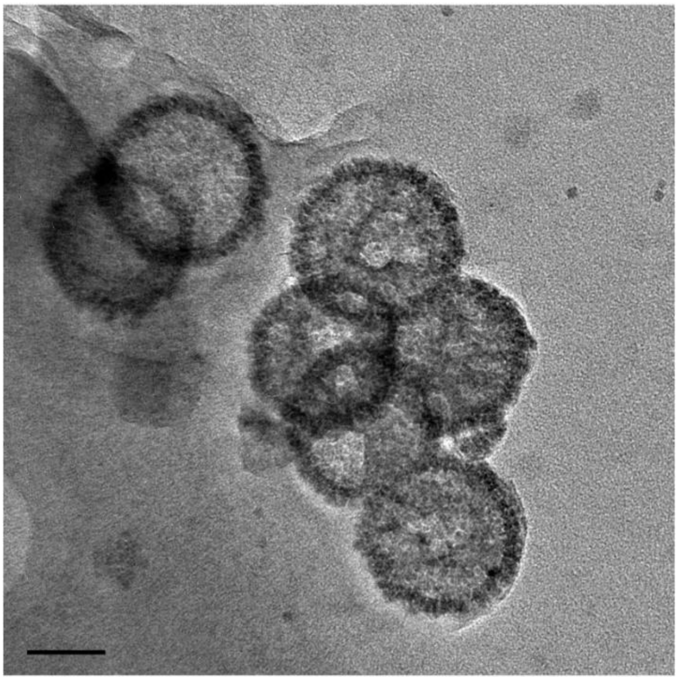

Supplementary Figure 15. TEM image of FHPG sample in 60 h of degradation in SBF (pH = 7.4). Scale bar, 50 nm. A representative image of three replicates is shown.

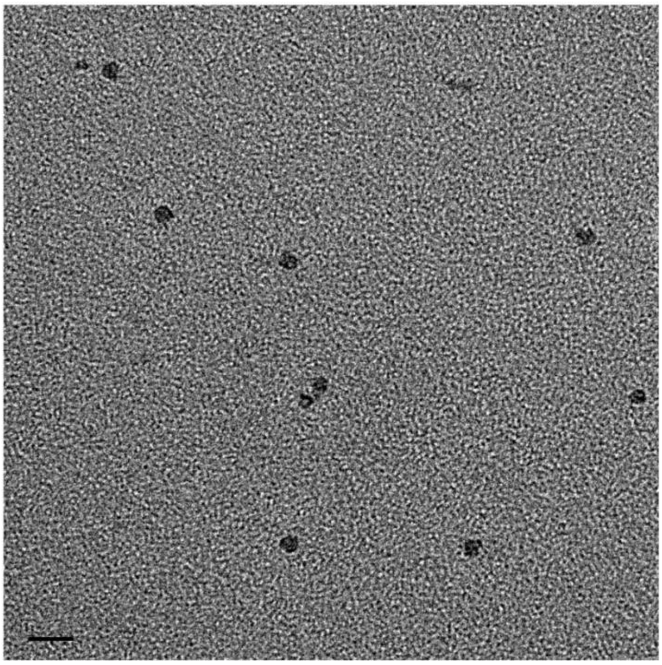

Supplementary Figure 16. TEM image of as-prepared fresh GA-Fe. Scale bar, 20 nm. A representative image of three replicates is shown.

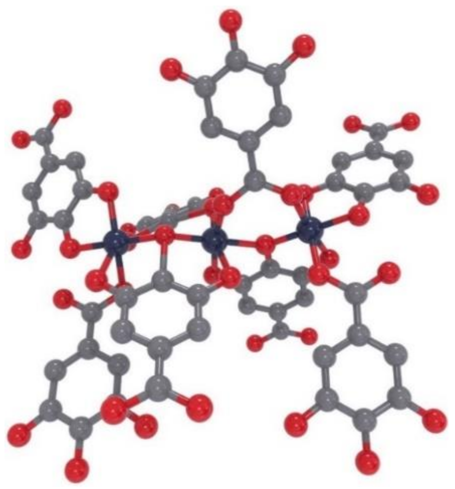

Supplementary Figure 17. Chemical structure for the tri-nuclear model of GA-Fe, $[Fe_3L_8H_{22}]^-$.

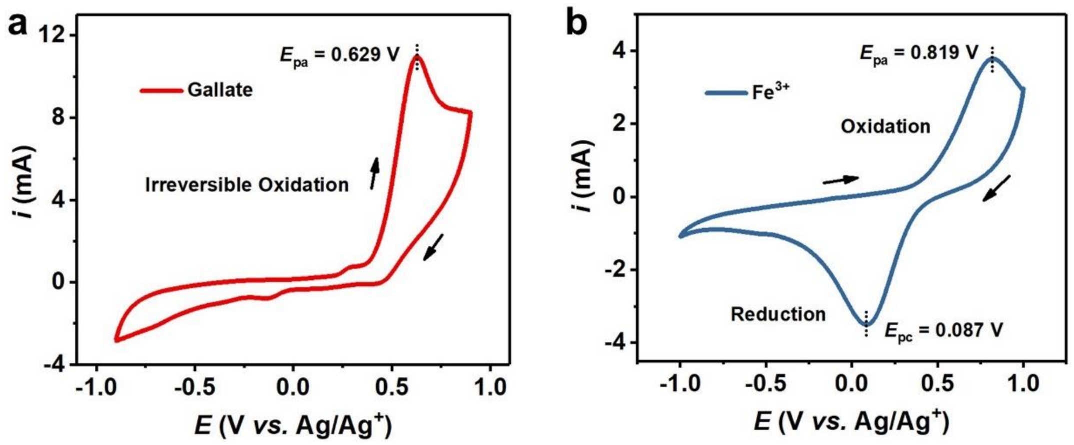

Supplementary Figure 18. CV curves for electrolyte solutions containing gallate **(a)** or Fe^{3+} **(b)**. Source data are provided as a Source Data file.

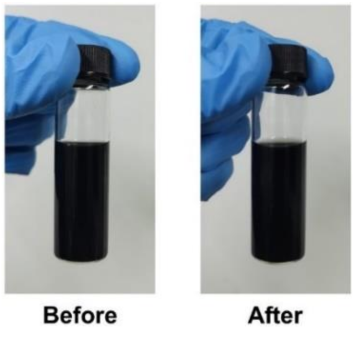

Supplementary Figure 19. Digital photos of GA-Fe-containing electrolyte solution before and after five circles of CV measurements.

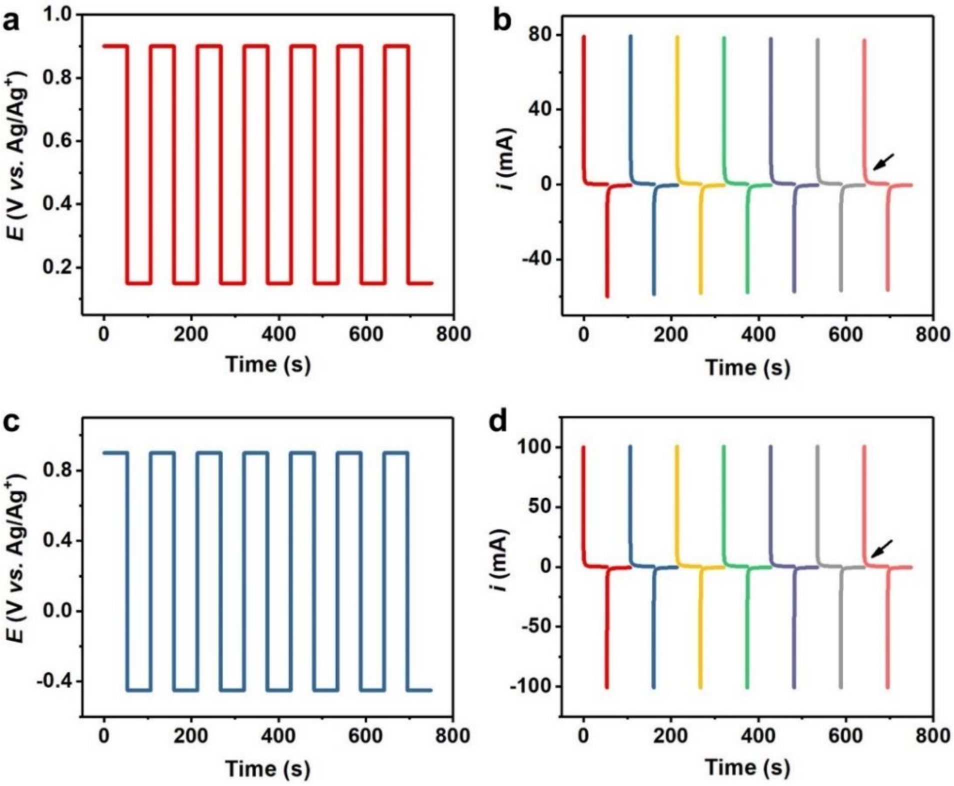

Supplementary Figure 20. (a) Seven consecutive periods of step potential transition (high potential: 0.90 V; low potential: 0.15 V) during CA measurement in Figure 5c. **(b)** CA profiles of electrolyte solution containing single Fe^{3+} responsive to consecutive step potential transitions in **(a)**. The limited current response is not changed during the seven periods of step potential transitions. **(c)** Seven consecutive periods of step potential transition (high potential: 0.90 V; low potential: -0.45 V) during CA measurement in Figure 5e. **(d)** CA profiles of electrolyte solution containing single Fe^{3+} responsive to consecutive step potential transitions in **(c)**. The limited current response is not changed during the seven periods of step potential transitions. According to Supplementary Figure 18b, E_{pc} (Fe^{3+}) = 0.087 V, Fe^{3+} could not be reduced during the periodic step potential transitions in **(a)** but was able to be reduced to Fe^{2+} during the periodic step potential transitions in **(c)** to enable the continuous Fe^{3+}/Fe^{2+} redox cycle. Source data are provided as a Source Data file.

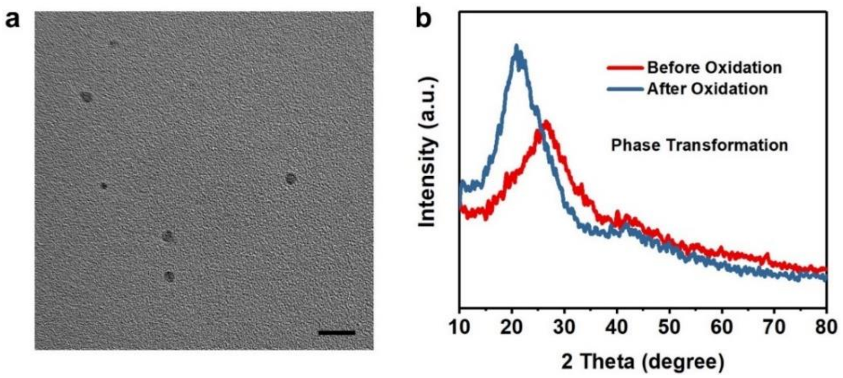

Supplementary Figure 21. (a) TEM image of GA-Fe nanoparticles after two rounds of CA measurements. Scale bar, 20 nm. A representative image of three replicates is shown. **(b)** XRD patterns of GA-Fe nanoparticles before and after electrochemical oxidation enabled by two rounds of CA tests. Source data are provided as a Source Data file.

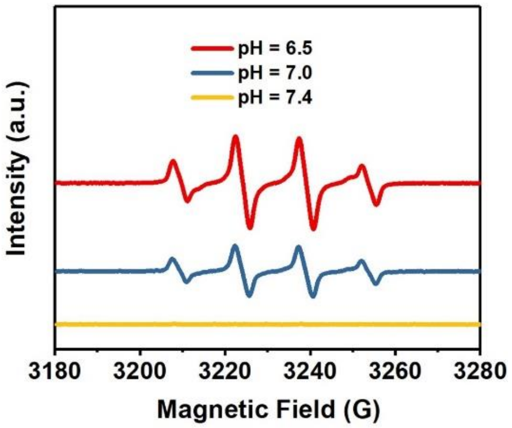

Supplementary Figure 22. ESR spectra evaluating •OH generation in buffer solutions of different pHs containing GA_{ox}-Fe and H_2O_2. Source data are provided as a Source Data file.

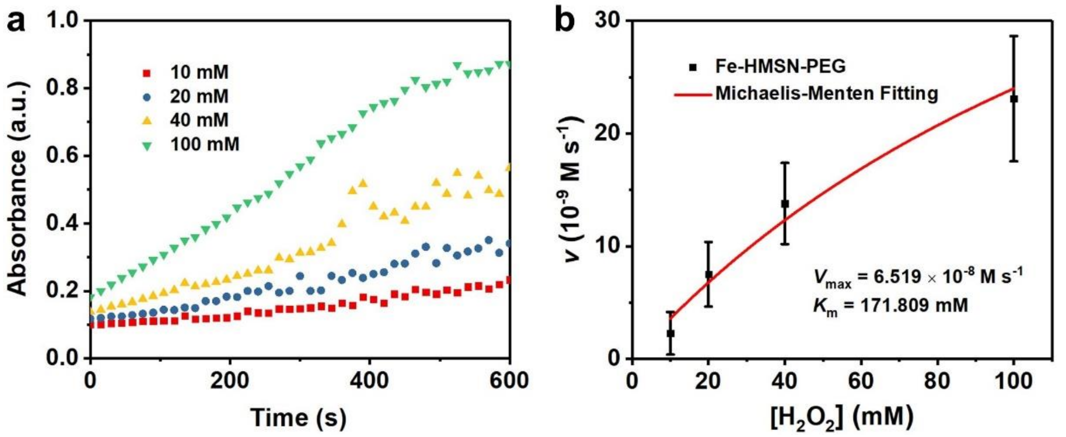

Supplementary Figure 23. **(a)** Time-course absorbance of buffer solution (pH = 6.5) containing Fe-HMSN-PEG and TMB after adding different concentrations of H_2O_2. **(b)** Michaelis–Menten kinetics of Fe-HMSN-PEG based on **(a)**. At one specific H_2O_2 concentration, the initial velocity of catalytic reaction was calculated by averaging the mean velocities of initial eight periods in **(a)** (15 s per period). Data are expressed as means ± SD (N = 8 independent experiments). Source data are provided as a Source Data file.

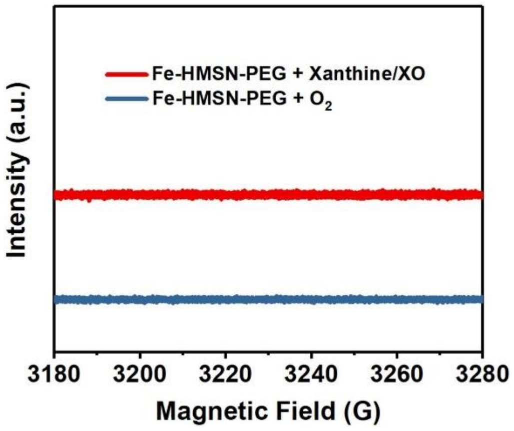

Supplementary Figure 24. ESR spectra evaluating •OH generation in buffer solution (pH = 6.5) containing Fe-HMSN-PEG under the presence of xanthine/XO ($O_2^{\bullet -}$ generator) or O_2. Source data are provided as a Source Data file.

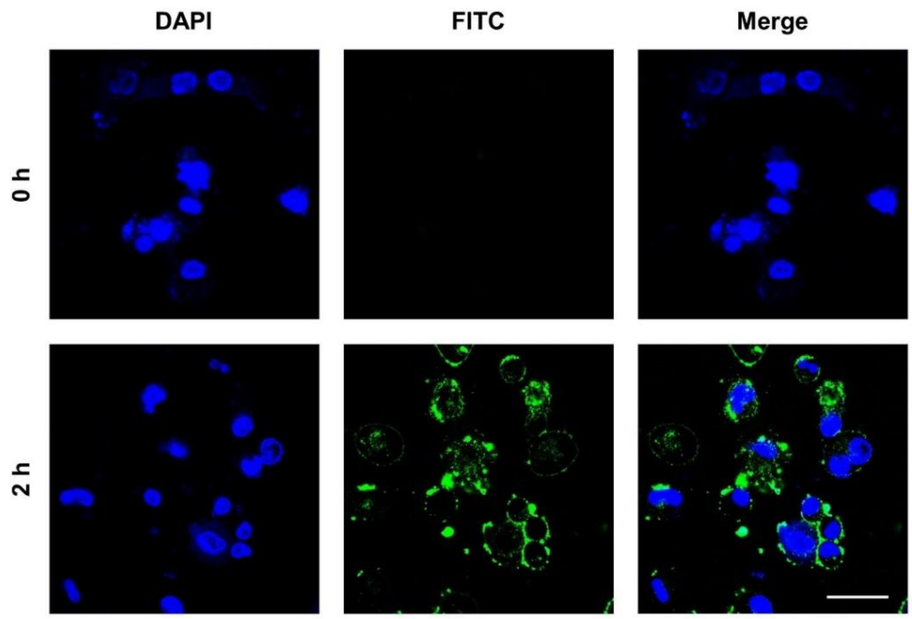

Supplementary Figure 25. CLSM images of HeLa cells after treated with FITC-labeled FHPG for 0 and 2 h. DAPI was used to stain cell nucleuses. Scale bar, 80 μm. A representative image of three replicates from each group is shown.

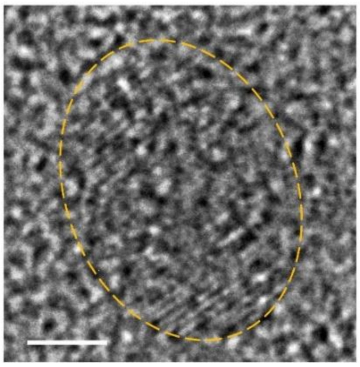

Supplementary Figure 26. High-resolution TEM image of a GA-Fe nanoparticle formed in HeLa cells. Scale bar, 2 nm. A representative image of three replicates is shown.

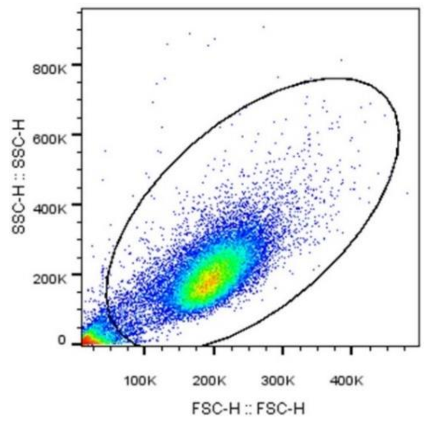

Supplementary Figure 27. Gating strategy to sort HeLa cells for flow cytometric studies in Figure 7d.

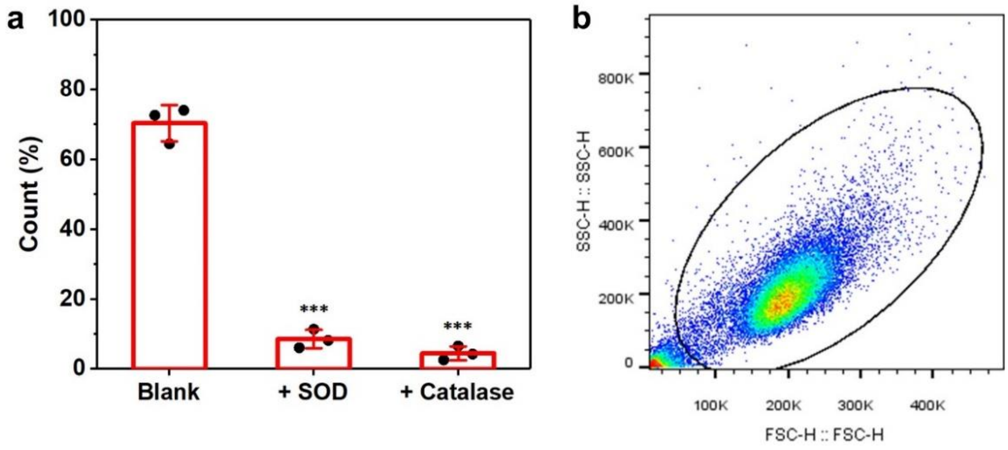

Supplementary Figure 28. (a) Quantitative flow cytometric analysis on the relative cellular •OH concentration of HeLa cells. To investigate cellular pro-oxidation process triggered by GA-Fe generation, primary HeLa cells were infected with adenovirus encoding SOD or catalase, respectively, then treated with FHPG for 6 h and stained with the •OH indicator DCFH-DA. Blank group indicates primary HeLa cells without adenovirus infection but with FHPG treatment. Data are expressed as means ± SD ($N = 3$ independent experiments). ***$P < 0.001$, based on the Student's two-sided *t*-test. **(b)** Gating strategy to sort HeLa cells for flow cytometric studies in **(a)**. Source data are provided as a Source Data file.

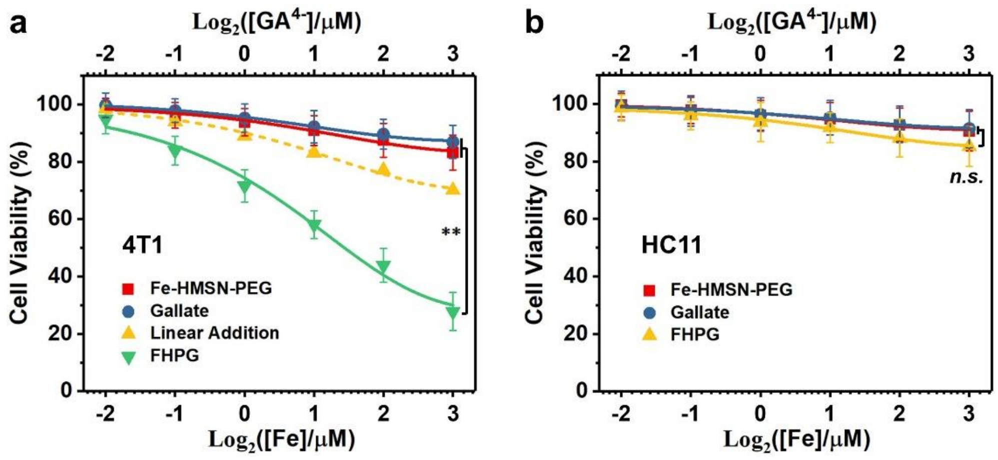

Supplementary Figure 29. Relative viabilities of 4T1 **(a)** and HC11 **(b)** cells after different treatments for 24 h. Linear addition for the effect of single Fe-HMSN-PEG or gallate treatment on relative viability of 4T1 cells is plotted for comparison with that of FHPG group. Data are expressed as means ± SD ($N = 6$ independent experiments). $^{**}P < 0.01$, *n.s.* is for not significant, based on the Student's two-sided *t*-test. Source data are provided as a Source Data file.

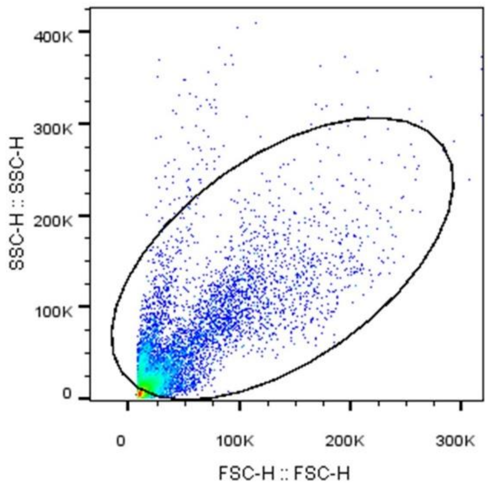

Supplementary Figure 30. Gating strategy to sort HeLa cells for flow cytometric studies in Figure 7h.

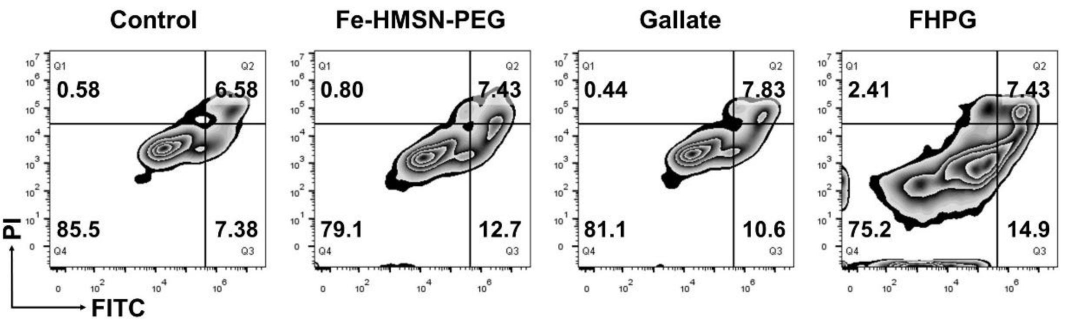

Supplementary Figure 31. Flow cytometry investigating the death mechanism of HUVECs after indicated treatments for 24 h, representative of three independent experiments.

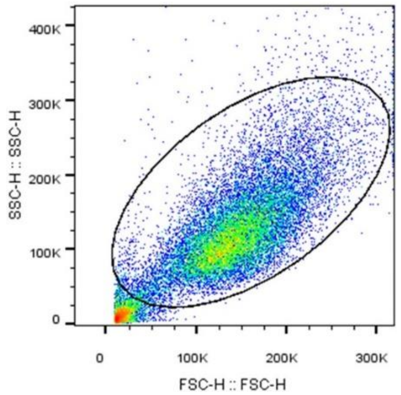

Supplementary Figure 32. Gating strategy to sort HUVECs for flow cytometric studies in Supplementary Figure 31.

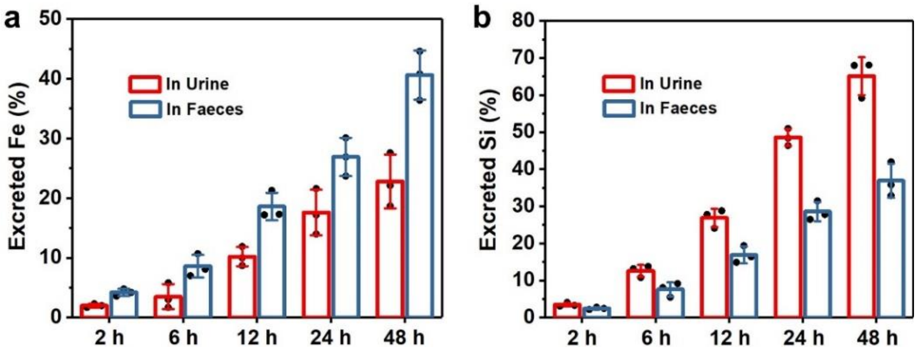

Supplementary Figure 33. Time-dependent Fe **(a)** and Si **(b)** excretions from HeLa tumor-bearing mice after FHPG administration. Data are expressed as means ± SD ($N = 3$ biologically independent animals). Source data are provided as a Source Data file.

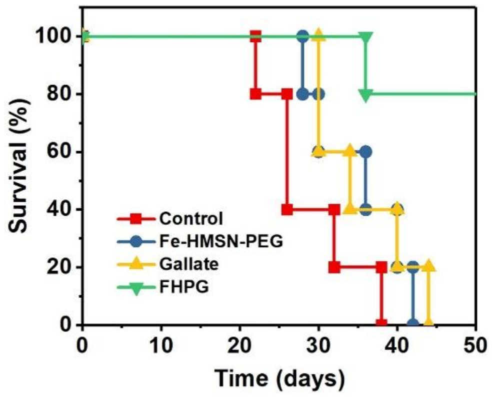

Supplementary Figure 34. Kaplan-Meier survival curves of HeLa tumor-bearing mice during 50 days of observation (*N* = 5 biologically independent animals). Source data are provided as a Source Data file.

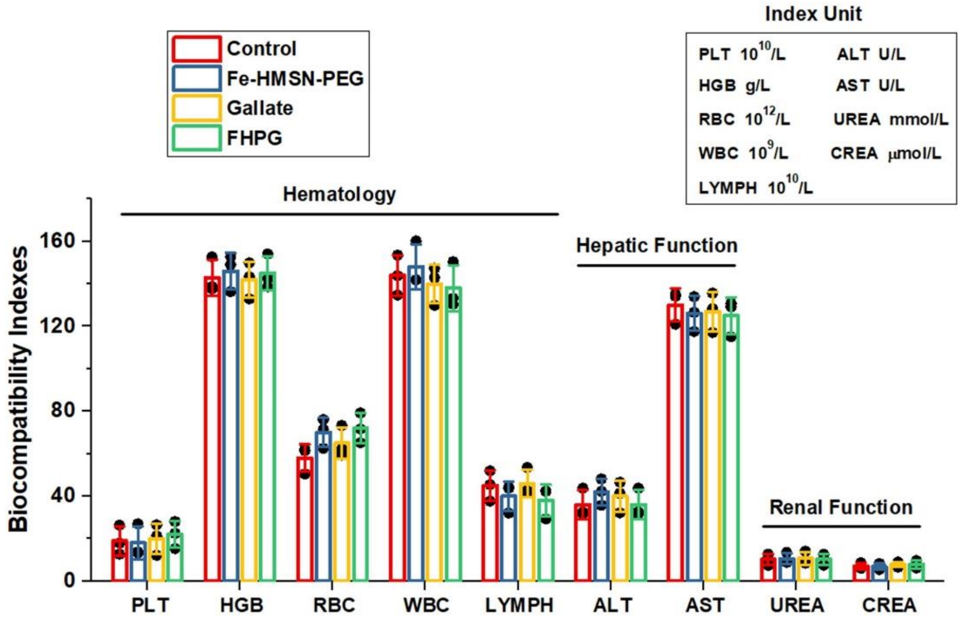

Supplementary Figure 35. Several key hematological, hepatic, and renal parameters of HeLa tumor-bearing mice after different treatments on day 28. Data are expressed as means ± SD (*N* = 3 biologically independent animals). Source data are provided as a Source Data file.

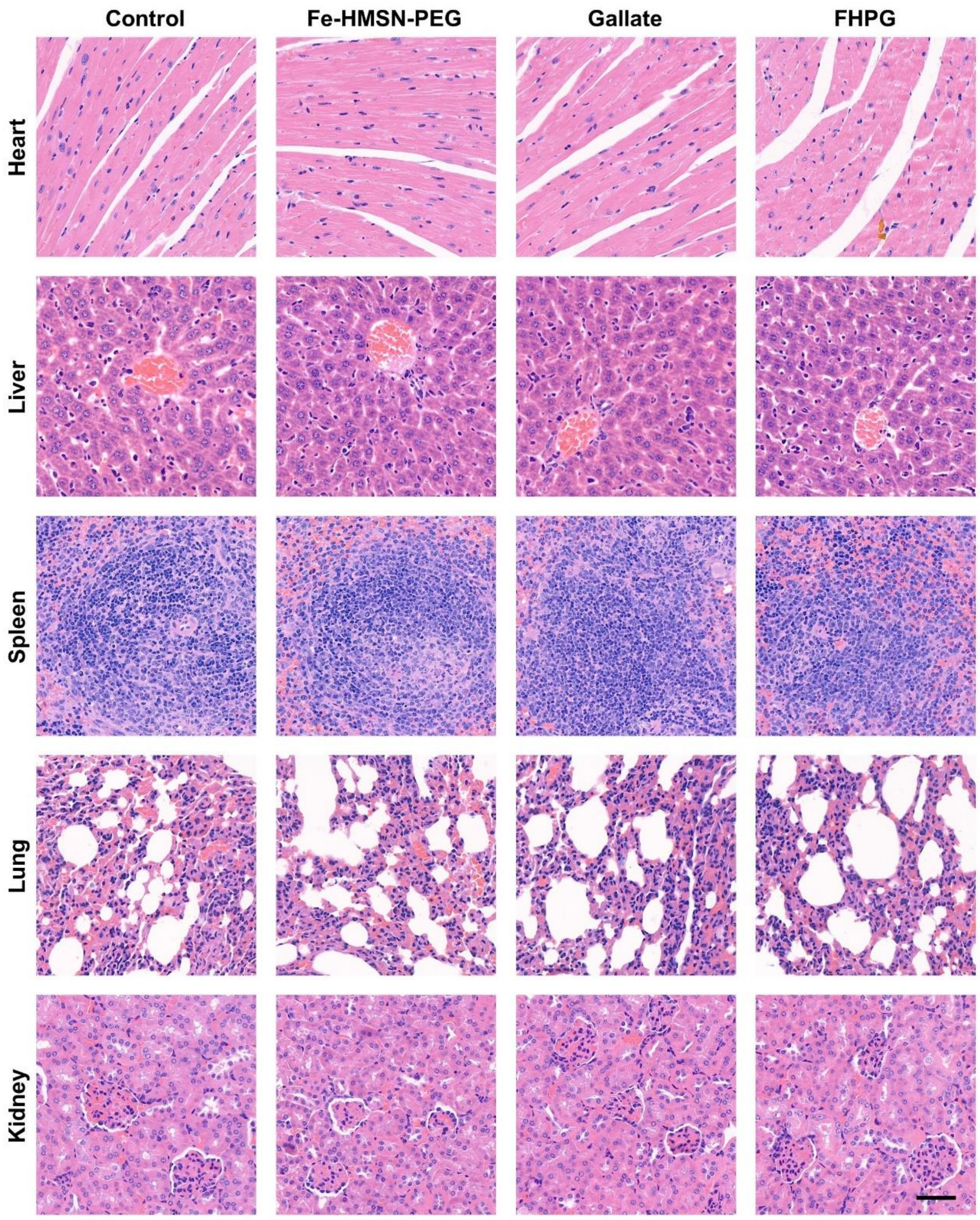

Supplementary Figure 36. H&E staining of major organs (hearts, livers, spleens, lungs, and kidneys) harvested from HeLa tumor-bearing mice after various treatments on day 14, representative of five biological replicates from each experimental group. Scale bar, 50 μm.

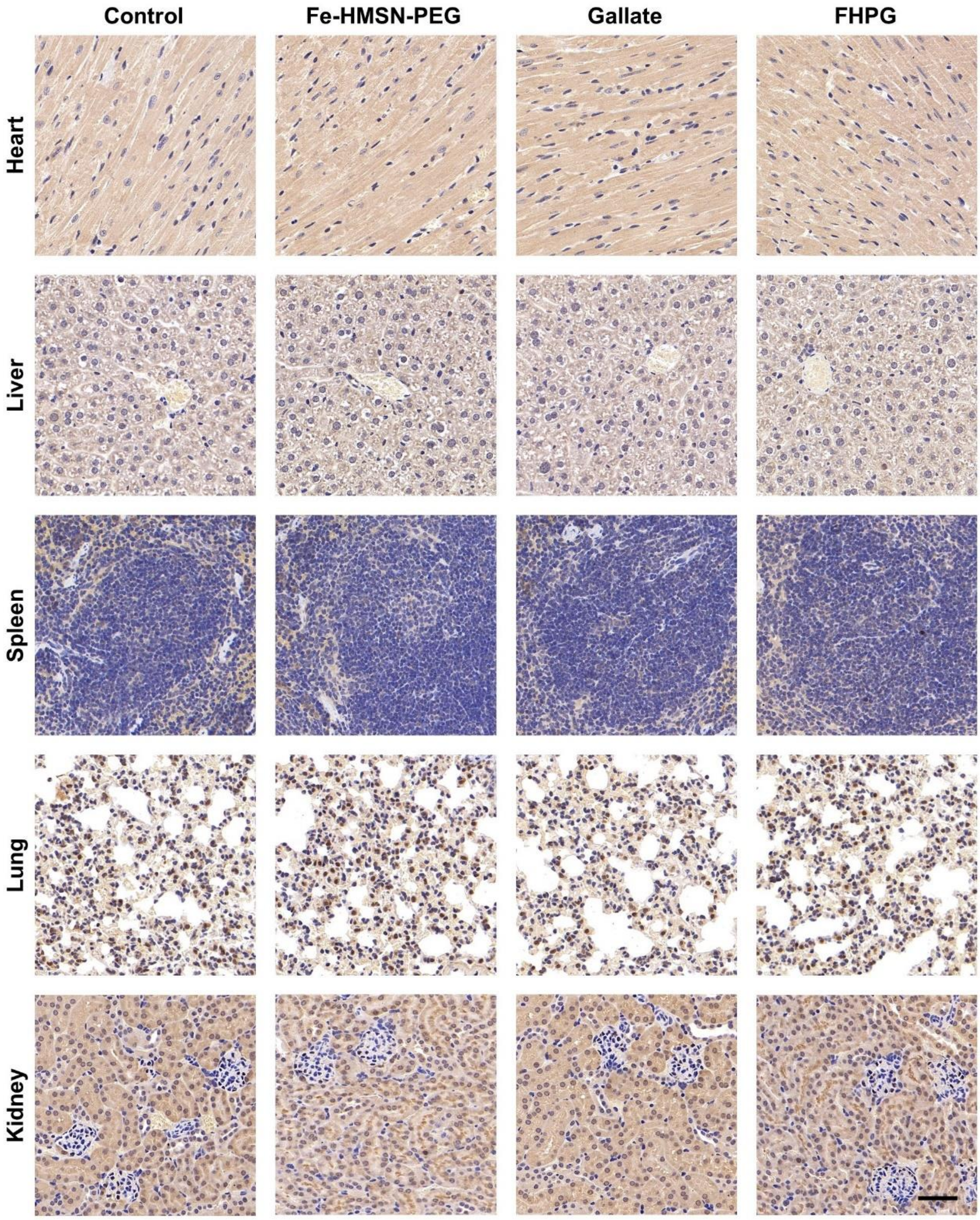

Supplementary Figure 37. GPX4 expressions in major organs (hearts, livers, spleens, lungs, and kidneys) harvested from HeLa tumor-bearing mice after various treatments on day 14, representative of five biological replicates from each experimental group. Scale bar, 50 μm.

Supplementary Tables

Supplementary Table 1. The differences between symmetric and asymmetric stretching vibrations ($\Delta\nu_{as\text{-}s}$) of -COO group of GA-Fe nanochelates, and their corresponding coordination modes of -COO group of gallate with Fe^{3+}.[1]

**$\Delta\nu_{as\text{-}s}$ (cm^{-1})**	> 168	< 168	<< 168
**Coordination Mode**	Monodentate 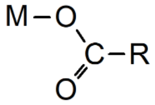	Bidentate 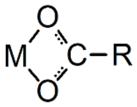	Bridge Type 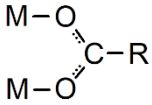

Supplementary Table 2. Crystal Data for ideal GA-Fe nanoparticles.

**Crystal Parameter**	**Data**
Formula	$[Fe(H_3O)(H_2O)(L)]_n$ ($L = GA^{4-}$)
Crystal system	Trigonal
Space group	$P3_121$
a (Å)	8.664
b (Å)	8.664
c (Å)	10.861
α (°)	90
β (°)	90
γ (°)	120
V (Å^3)	706.05

Supplementary Table 3. Atomic spin densities (M06/C-PCM calculations) for the tri-nuclear model $[Fe_3L_8H_{22}]^-$ (15 unpaired electrons) and its one-electron oxidation form $[Fe_3L_8H_{22}]^{2-}$ (14 unpaired electrons). Data are obtained from Bortoluzzi's work.[2] Population analyses were performed on the basis of the Mulliken and Hirshfeld distributions.[3, 4]

Atom	$[Fe_3L_8H_{22}]^-$		$[Fe_3L_8H_{22}]^{2-}$	
	Mulliken	Hirshfeld	Mulliken	Hirshfeld
Fe	4.1832	4.1393	4.1001	4.0312
O (Fe-O carboxylate)	0.0860	0.0940	0.0666	0.0781
O (Fe-O phenate)	0.1628	0.1615	0.1303	0.1293
O (not coordinated)	0.0021	0.0024	0.0005	0.0000
C (carboxylate)	0.0056	0.0080	-0.0179	-0.0019
C (phenate)	0.0047	0.0058	0.0025	0.0031
H(CH)	-0.0002	——	0.0003	——
H(OH)	0.0001	——	0.0001	——

Supplementary Table 4. Chemical reactions during CV measurement in Figure 5a ($\iiint dxdydz = n$).

**Negative Scanning**	$[Fe(III)GA_{ox}]^{+}_{(x,y,z)} + ne^{-} = [Fe(II)GA_{ox}]_{(x,y,z)}$ (15)
	$[Fe(III)GA\cdot]_{(x,y,z)} + ne^{-} = [Fe(II)GA\cdot]^{-}_{(x,y,z)}$ (16)
**Positive Scanning**	$[Fe(III)GA]^{-}_{(x,y,z)} \leftrightarrow [Fe(II)GA\cdot]^{-}_{(x,y,z)}$ (17)
	$[Fe(II)GA\cdot]^{-}_{(x,y,z)} - ne^{-} = [Fe(III)GA\cdot]_{(x,y,z)}$ (18)
	$[Fe(III)GA\cdot]_{(x,y,z)} \leftrightarrow [Fe(II)GA_{ox}]_{(x,y,z)}$ (19)
	$[Fe(II)GA_{ox}]_{(x,y,z)} - ne^{-} = [Fe(III)GA_{ox}]^{+}_{(x,y,z)}$ (20)

Supplementary Table 5. Chemical reactions during CA measurement in Figure 5c ($\iiint dxdydz = n$).

**Cathode**	—
**Anode**	$[Fe(III)GA]^{-}_{(x,y,z)} \leftrightarrow [Fe(II)GA\,\cdot]^{-}_{(x,y,z)}$ (21)<br>$[Fe(II)GA\,\cdot]^{-}_{(x,y,z)} - ne^{-} = [Fe(III)GA\,\cdot]_{(x,y,z)}$ (22)<br>$[Fe(III)GA\,\cdot]_{(x,y,z)} \leftrightarrow [Fe(II)GA_{ox}]_{(x,y,z)}$ (23)<br>$[Fe(II)GA_{ox}]_{(x,y,z)} - ne^{-} = [Fe(III)GA_{ox}]^{+}_{(x,y,z)}$ (24)<br>$[Fe(III)GA_{ox}]^{+}_{(x,y,z)} \leftrightarrow [Fe(II)GA_{ox}\,\cdot]^{+}_{(x,y,z)}$ (25)<br>$[Fe(II)GA_{ox}\,\cdot]^{+}_{(x,y,z)} - ne^{-} = [Fe(III)GA_{ox}\,\cdot]^{2+}_{(x,y,z)}$ (26)

Supplementary Table 6. Chemical reactions during CA measurement in Figure 5e ($\iiint dxdydz = n$).

**Cathode**	$[Fe(III)GA_{ox}\,\cdot]^{2+}_{(x,y,z)} + ne^{-} = [Fe(II)GA_{ox}\,\cdot]^{+}_{(x,y,z)}$ (27)<br>$[Fe(II)GA_{ox}\,\cdot]^{+}_{(x,y,z)} \leftrightarrow [Fe(III)GA_{ox}]^{+}_{(x,y,z)}$ (28)
**Anode**	$[Fe(III)GA_{ox}]^{+}_{(x,y,z)} \leftrightarrow [Fe(II)GA_{ox}\,\cdot]^{+}_{(x,y,z)}$ (29)<br>$[Fe(II)GA_{ox}\,\cdot]^{+}_{(x,y,z)} - ne^{-} = [Fe(III)GA_{ox}\,\cdot]^{2+}_{(x,y,z)}$ (30)

Supplementary Table 7. Accumulated Fe element release from GA-Fe nanoparticles after 7 circles of voltage steps during CA measurements in Figure 5c and 5e, determined by ICP-OES.

**Figure 5c (%)**	**Figure 5e (%)**
5.612	6.734

Supplementary Table 8. Comparison of the Michaelis constant (K_m) of different catalysts.

**Catalysts**	**K_m (mM)**	**Ref**
Fe_3O_4 Nanoparticles	154	5
HRP	3.70	5
GA_{ox}-Fe	24.81	This work
Fe-HMSN-PEG	171.81	This work

Supplementary References

1. Nakamoto, K. *Infrared and Raman Spectra of Inorganic and Coordination Compounds (Part B)*. (John Wiley, 1997).
2. Zaccaron, S., Ganzerla, R. & Bortoluzzi, M. Iron complexes with gallic acid: a computational study on coordination compounds of interest for the preservation of cultural heritage. *J. Coord. Chem.* **66**, 1709-1719 (2013).
3. Mulliken, R. S. Electronic Population Analysis on LCAO–MO Molecular Wave Functions. I. *J. Chem. Phys.* **23**, 1833-1840 (1955).
4. Hirshfeld, F. L. Bonded-Atom Fragments for Describing Molecular Charge-Densities. *Theor. Chim. Acta* **44**, 129-138 (1977).
5. Gao, L., Zhuang, J., Nie, L., Zhang, J., Zhang, Y., Gu, N. *et al.* Intrinsic peroxidase-like activity of ferromagnetic nanoparticles. *Nat. Nanotechnol.* **2**, 577-583 (2007).
